# Successful biliary decompression for multiple biliary obstructions by bridging stenting using the partial stent-in-stent method via endoscopic ultrasound-guided hepaticogastrostomy

**DOI:** 10.1055/a-2559-4221

**Published:** 2025-03-25

**Authors:** Masaki Miyazawa, Masaki Nishitani, Noriaki Orita, Tomoyuki Hayashi, Shinya Yamada, Hajime Takatori, Taro Yamashita

**Affiliations:** 1 88335 Department of Gastroenterology, Kanazawa University Hospital, Kanazawa, Japan

Recently, the usefulness of endoscopic ultrasound-guided hepaticogastrostomy (EUS-HGS) for malignant hilar biliary obstruction (MHBO) has been reported, offering a feasible alternative approach in cases where transpapillary stenting is impossible 1 2 3 4 . Herein, we present a case in which bridging stenting using the partial stent-in-stent method via the EUS-HGS route was performed to treat multiple right intrahepatic bile duct (IHBD) obstructions ( Video 1 ).

Bridging placement of two uncovered self-expandable metal stents in B5 and B8 using the partial stent-in-stent method via the endoscopic ultrasound-guided hepaticogastrostomy route.Video 1

A 53-year-old woman who had undergone transpapillary stenting using uncovered self-expandable metal stents (UCSEMSs) for MHBO due to cancer of unknown primary developed cholangitis because of tumor ingrowth in the UCSEMSs. Transpapillary stenting was impossible due to tumor invasion into the duodenum. Therefore, we performed EUS-HGS, and a partially covered SEMS (PCSEMS) with anchoring properties was deployed in the left IHBD B3 ( Fig. 1 **a** ). Eleven months later, the patient presented with fever and elevated serum bilirubin levels, and was diagnosed with cholangitis accompanied by progressive dilatation of the right IHBDs B5 and B8, the cause of which was thought to be tumor overgrowth ( Fig. 1 **b, c** ). We planned to place two UCSEMSs in B5 and B8 using the partial stent-in-stent method via the EUS-HGS route.

**Fig. 1:**
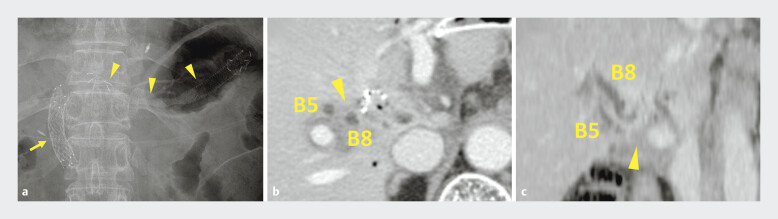
**a** Abdominal X-ray image shows uncovered self-expandable metal stents in the hilar bile duct placed via a transpapillary approach (arrow) and a partially covered self-expandable metal stent deployed in B3 using endoscopic ultrasound-guided hepaticogastrostomy (arrowheads). **b, c** Computed tomography images show tumor overgrowth toward (arrowhead) the liver and dilated intrahepatic bile ducts (B5 and B8).

First, the cover of the PCSEMS was broken through ( Fig. 2 **a** ), and two guidewires were inserted through the overgrowth into the dilated B5 and B8 ( Fig. 2 **b, c** ). Using a movable-tip cannula (Zeon Medical Inc., Tokyo, Japan) and a tapered cannula (PR-110Q-1; Olympus, Tokyo, Japan), we devised a way for the delivery system to break through the overgrowth and the stent mesh, and ultimately placed two UCSEMSs (Zeo stent V; Zeon Medical Inc.) in B8 and then B5 with the partial stent-in-stent method. After the procedure, the patient’s cholangitis showed improvement and has not recurred for 14 months.

**Fig. 2:**
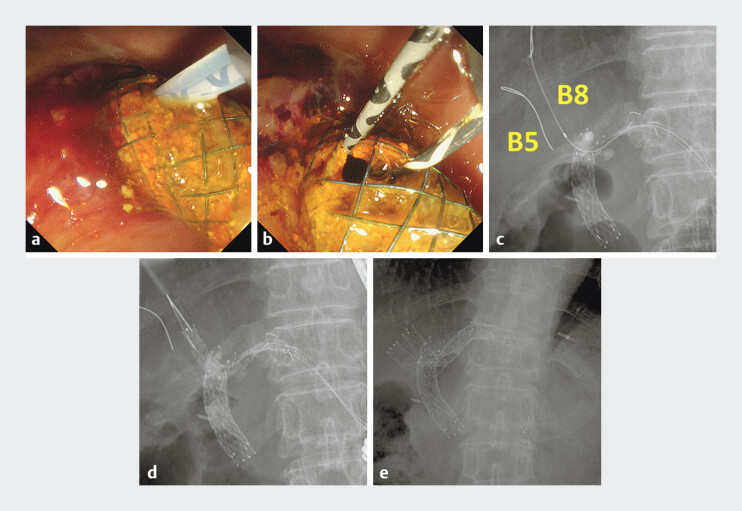
**a** The cover of the partially covered self-expandable metal stent was broken through by the canula. **b, c** Two guidewires were inserted through the overgrowth into the dilated B5 and B8. **d, e** Two uncovered self-expandable metal stents were placed in B5 and B8, respectively, using the partial stent-in-stent method.

We believe that this multiple bridging stenting is effective for biliary decompression of the right lobe when transpapillary stenting is not possible.

Endoscopy_UCTN_Code_TTT_1AS_2AH

## Notes

Endoscopy E-Videos https://eref.thieme.de/e-videos
*E-Videos* is an open access online section of the journal *Endoscopy* , reporting on interesting cases and new techniques in gastroenterological endoscopy. All papers include a high-quality video and are published with a Creative Commons CC-BY license. Endoscopy E-Videos qualify for HINARI discounts and waivers and eligibility is automatically checked during the submission process. We grant 100% waivers to articles whose corresponding authors are based in Group A countries and 50% waivers to those who are based in Group B countries as classified by Research4Life (see: https://www.research4life.org/access/eligibility/ ). This section has its own submission website at https://mc.manuscriptcentral.com/e-videos .
